# New insights on *mcr-1*-harboring plasmids from human clinical *Escherichia coli* isolates

**DOI:** 10.1371/journal.pone.0294820

**Published:** 2024-02-26

**Authors:** Florencia Martino, Alejandro Petroni, María Alejandra Menocal, Alejandra Corso, Roberto Melano, Diego Faccone

**Affiliations:** 1 Servicio Antimicrobianos, National Reference Laboratory in Antimicrobial Resistance (NRLAR), National Institute of Infectious Diseases (INEI), ANLIS “Dr. Carlos G. Malbrán”, Buenos Aires City, Argentina; 2 Consejo Nacional de Investigaciones Científicas y Técnicas (CONICET), Buenos Aires City, Argentina; 3 Public Health Ontario Laboratory, Toronto, Ontario, Canadá; 4 University of Toronto, Toronto, Ontario, Canadá; 5 Pan American Health Organization, Washington, D.C., United States of America; North Carolina State University, UNITED STATES

## Abstract

Mobile colistin resistance (*mcr*) genes were described recently in Gram-negative bacteria including carbapenem-resistant Enterobacterales. There are ten *mcr* genes described in different Gram-negative bacteria, however, *Escherichia coli* harboring *mcr-1* gene is by far the most frequent combination. In Argentina, *mcr-1* gene was characterized only on plasmids belonging to IncI2 group. The aim of this work was to get new insights of *mcr-1*-harboring plasmids from *E*. *coli*. Eight *E*. *coli* isolates from a larger collection of 192 clinical *E*. *coli* isolates carrying the *mcr-1* gene were sequenced using next generation technologies. Three isolates belonged to ST131 high-risk clone, and five to single ST, ST38, ST46, ST226, ST224, and ST405. Eight diverse *mcr-1*-harboring plasmids were analyzed: IncI2 (1), IncX4 (3), IncHI2/2A (3) and a hybrid IncFIA/HI1A/HI1B (1) plasmid. Plasmids belonging to the IncI2 (n = 1) and IncX4 (n = 3) groups showed high similarity with previously described plasmids. Two IncHI2/HI2A plasmids, showed high identity between them, while the third, showed several differences including additional resistance genes like *tet(A)* and *floR*. One IncFIA/H1A/H1B hybrid plasmid was characterized, highly similar to pSRC27-H, a prototype plasmid lacking *mcr* genes. *mcr-1*.*5* variant was found in four plasmids with three different Inc groups: IncI2, IncHI2/HI2A and the hybrid FIA/HI1A/HI1B plasmid. *mcr-1*.*5* variant is almost exclusively described in our country and with a high frequency. In addition, six *E*. *coli* isolates carried three allelic variants codifying for CTX-M-type extended-spectrum-β-lactamases: *bla*_CTX-M-2_ (3), *bla*_CTX-M-65_ (2), and *bla*_CTX-M-14_ (1). It is the first description of *mcr-1* harboring plasmids different to IncI2 group in our country. These results represents new insights about *mcr-1* harboring plasmids recovered from *E*. *coli* human samples from Argentina, showing different plasmid backbones and resistance gene combinations.

## Introduction

Polymyxins, including polymyxin B and colistin, are “last-line” treatment options against multidrug-resistant Gram-negative bacteria such as carbapenem-resistant *Enterobacterales*. Until November 2015, the main colistin resistance mechanisms reported were based on chromosomal mutations involving alterations in the two-component regulatory systems PmrAB, or PhoPQ [1]. The scenario changed with the report of mobile colistin resistance (*mcr*) mediated by *mcr-1* gene revealing for the first time the horizontal spread of a colistin resistance determinant [1]. This gene encodes a plasmid-borne phosphoethanolamine transferase and has been worldwide reported in different Gram-negative bacteria, being *Escherichia coli* the main reported species, although it has also been detected in other *Enterobacterales*, including *Klebsiella pneumoniae* and *Salmonella* spp. [2]. *mcr-1*-harboring isolates were recovered from human samples corresponding to both infection and colonization events, and also from animals, food and the environment [2]. Up to now, ten *mcr* gene types have been described (*i*.*e*., *mcr-1* to *mcr-10*) and several alleles were reported for most of them, for example, *mcr-1*.*1* to *mcr-1*.*34* for *mcr-1* type, which is the most widespread worldwide by far [3, 4]. In particular, *mcr-1* has been described in almost all the American continent, while *mcr-3* and *mcr-5* were sporadically described in Brazil and Colombia, respectively [5, 6]. Isolates containing *mcr* genes have also been identified in multidrug- and extensively-resistant strains, some of them expressing additional concerning antimicrobial resistance genes such as carbapenemases and extended-spectrum β-lactamases [5, 7].

*mcr-1* is mostly located in Tn*6330* composite transposon, which comprises two IS*Apl1* insertion sequences flanking a 2607 bp-long DNA fragment containing *mcr-1* (1626 bp) and *pap2* (765 bp) genes [8, 9]. It was proposed that sometimes after the insertion of Tn*6330* in a new target, the insertion sequences IS*ApI1* could be lost and give rise to *mcr-1* genes immobilized in different plasmid backgrounds [8]. In some circumstances, a single copy of IS*Apl1*, upstream of *mcr-1*, together with the remnants of the IS*Apl1* located downstream of that gene, could also mobilize *mcr-1* [8]. *mcr-1* has been found in a variety of different incompatibility (Inc) group plasmids, being IncI2 and IncX4 followed by IncHI2 the most worldwide described plasmids [9].

IncI2 *mcr-1*-harboring plasmids range in size 60–62 kb with an average GC content of 42–43% [10]. The IncI2 backbone encodes replication, horizontal transfer, maintenance and stability functions [10, 11]. These plasmids harbor a multiple DNA inversion system, named shufflon, composite of three (A, BD and C) segments which can rearrange among them and generate different PilV tip adhesins, which recognize specific lipopolysaccharide (LPS) structures [11]. Therefore, the shufflon rearrangement would be related to plasmid transmission to a broad range of Enterobacterales [11]. *mcr-1*-containing IncI2 plasmids harbor this gene as the only resistance mechanism [10, 11]. Generally, Tn*6330* insertions in IncI2 plasmids occurred at a conserved palindromic sequence located immediately downstream of *nikB* gene [12]. IncX4 plasmids are self-transmissible and in general the size of them range 33–35 kb with a GC content of 39–42% [10, 13]. It was noticed that in *mcr-1*-containing IncX4 plasmids, both IS*ApI1* copies were lost yielding a Tn*6330* remnant structure [10, 13]. *mcr-1*-containing IncX4 plasmids share high conserved backbone sequences among them [10, 13]. Plasmids belonging to the IncHI complex can be subclassified into three incompatibility groups, IncHI1, IncHI2 and IncHI3 [14]. The IncHI plasmids are phylogenetically related and share common regions coding for conjugative functions, plasmid replication and maintenance, plus others functions like ultraviolet light protection, or thermoregulation of conjugation [14]. In particular, the largest *mcr-1*-harboring plasmids described to date belong to IncHI2A, with sequence lengths around 270 kb [15]. Finally, hybrid plasmids containing replicases belonging to more than one incompatibility group are rare, and are likely a consequence of IS-mediated fusions or homologous recombination events [16, 17]. The presence of multiple incompatibility groups increases the host range of each single hybrid plasmid, rising the dissemination potential of antimicrobial resistance [16].

Regardless of the source of isolation, the most commonly *mcr-1*-harboring plasmids described across the Americas belonged to IncI2 group, being described in at least 13 countries: Argentina, Bolivia, Brazil, Canada, Chile, Colombia, Ecuador, Mexico, Paraguay, Peru, USA, Uruguay and Venezuela [5]. IncX4 plasmids were only described in four countries: Argentina, Brazil, USA and Uruguay, while plasmids belonging to IncHI1, IncHI2, IncFIB, IncFII, IncP-1 and IncF8:A-:B1 groups were sporadically described in the Americas [5]. IncI2 and IncX4 *mcr-1*-harboring plasmids usually had no additional resistance genes contrarily to the other plasmid groups [11, 13]. Therefore, the study of *mcr-1*-harboring plasmids that do not belong to IncI2 and IncX4 groups is also very relevant for the epidemiology of other resistance genes and the potential of co-selection of different resistance mechanisms. Few reports from the Americas included closed plasmid sequences, limiting a broad understanding about *mcr-1* dissemination across this region. In Argentina only few *mcr-1*-harboring plasmids recovered from clinical Enterobacterales were fully characterized belonging to IncI2 group [18–22]. Therefore, the study of more closed sequences of *mcr-1*-harboring plasmids could provide new insights for the epidemiology of this relevant antimicrobial resistance gene in both Argentina and the region of the Americas. Indeed, in a previous work, we studied a collection of 192 *mcr-1*-harboring *E*. *coli* clinical isolates, recovered from 69 hospitals of Buenos Aires City and 14 Argentinian provinces, between 2012 and 2018 [23]. All 192 *E*. *coli* isolates showed MICs of colistin ≥ 4μg/mL, and nearly 50% were resistant to extended-spectrum cephalosporins, being CTX-M-2 the main extended-spectrum β-lactamase detected. Five *E*. *coli* were carbapenemase-producers: 3 NDM- and 2 KPC-type enzymes [23]. No genetic relationship among 110 out of 192 *mcr-1*-positive *E*. *coli* was observed by XbaI-PFGE. The *mcr-1*.*5* variant was found in 13.5% of the 192 *E*. *coli* isolates by using an allele-specific PCR to detect this particular *mcr* variant [23]. Additionally, a high proportion (164/192; 85%) of IncI2 group, and a low frequency of IncX4 (18/192; 9.4%) was observed by using a PCR plasmid typing. These results showed that IncI2 and IncX4 are the most common replicon types in *mcr-1*-harboring isolates. Finally, only 10 isolates (5.2%) were negative for both IncI2 and IncX4 groups [23]. Herein, we obtained and analyzed more closed sequences of *mcr-1*-harboring plasmids that belonged not only to IncI2 and IncX4 but also IncHI2/HI2A, as well as an FIA/HI1A/HI1B hybrid plasmid.

## Materials and methods

In a previous study, we characterized a collection of 192 *mcr-1*-harboring *E*. *coli* clinical isolates, which were collected in 69 hospitals of Buenos Aires City and 14 Argentinian provinces, between 2012 and 2018 (see epidemiological data in [23]). These isolates were submitted to the NRLAR as part of the national antimicrobial resistance surveillance and did not include human biological material nor patient identification. Therefore ethics approval was not required for this study. This collection of 192 *mcr-1*-harboring *E*. *coli* was used to select eight isolates for further characterization, aimed to better comprehend the *mcr-1* landscape dissemination in our region. The isolates were purposively chosen in order to cover several epidemiological aspects as hospital diversity, isolation date, sequence typing, Inc group, and *bla*_CTX-M_ variants. This selection included: i) one IncI2-positive isolate belonging to ST131 and collected more recently than IncI2 plasmid-harboring isolates previously characterized [18]); ii) three IncX4-positive isolates carrying different *bla*_CTX-M_ variants (i.e. *bla*_CTX-M-2_, *bla*_CTX-M-9/14_, and *bla*_CTX-M-8/25_); and iii) four isolates negative for both IncI2- and IncX4-groups, three of which also harbored *bla*_CTX-M-2_ (2) or *bla*_CTX-M-9/14_ (1) variants. These eight isolates were recovered from five specimens (urine, blood, stool culture, abscess and bone), collected in eight hospitals located in four provinces and Buenos Aires City (Table 1). Whole bacterial DNA was extracted with QIAcube, using the QIAamp^®^ DNA Mini Kit (Qiagen) for short read sequencing (MiSeq, Illumina), and MasterPure^™^ Complete DNA&RNA Purification Kit (Lucigen, Epicentre) for long read sequencing (MinION, Oxford Nanopore Technologies).

**Table 1:** Epidemiological and genomic information of eight *E*. *coli* harboring *mcr-1* gene.

**Epidemiological Information**								
Strain ID	M22546	M21816	M23312	M23370	M21170	M23314	M23059	M23917
Isolation Date	21-Jul-17	23-Nov-16	12-Apr-18	27-Jun-18	7-Apr-16	23-May-18	24-Oct-17	30-Dec-18
Hospital	H1	H2	H3	H4	H5	H6	H7	H8
Province	Cordoba	CABA	CABA	CABA	Mendoza	La Pampa	Jujuy	CABA
Sample	urine	abscess	bone	blood	urine	blood	stool	urine
**Genomic Infromation**								
Sequence type (ST)	131	131	131	38	405	224	226	46
Clonal Complex	CC131	CC131	CC131	CC38	CC405	-	CC226	CC46
***mcr-1*-harboring plasmids**								
Name	pEco_M22546_62	pEco_M21816_274	pEco_M23312_272	pEco_M23370_33	pEco_M21170_247	pEco_M23314_185	pEco_M23059_33	pEco_M23917_34
Size (bp)	62,883	274,233	272,608	33,304	247,558	185,802	33,304	34,504
Incompatibility group	I2	HI2/HI2A	HI2/HI2A	X4	HI2/HI2A	FIA/HI1A/HI1B	X4	X4
Additional antimicrobial resistance genes	-	*qacE*, *bla*_*CTX-M-2*_, *aadA1*, *mph(B)*, *sul1*, *sat2*	*qacE*, *bla*_*CTX-M-2*_, *aadA1*, *mph(B)*, *sul1*, *sat2*	-	*tet(A)*, *floR*	*sul3*, *aadA2b*, *aadA2*, *cmlA1*, *qacL*	-	-
*mcr-1* alelle	*mcr-1*.*5*	*mcr-1*.*5*	*mcr-1*.*5*	*mcr-1*.*1*	*mcr-1*.*1*	*mcr-1*.*5*	*mcr-1*.*1*	*mcr-1*.*1*
**Other antimicrobial resistance genes**								
Other antimicrobial resistance genes in other plasmids and/or chromosome	-	*tet(A)*, *dfrA17*, *aadA5*, *sul2*	-	*sul1*, *bla*_CTX-M-2_, *aadA5*, *dfrA17*, *aph(3’’)-Iia*	*bla*_CTX-M-14*_, *aadA1*, *sat2*, *bla*_TEM-1_, *tet(B)*, *aph(3’’)-Ib*, *aph(6)-Id*	*blaTEM-1*, *tet(a)*	*bla*_TEM-1_, *aac(3)-Iva*, *aph(4)-Ia*, *floR*, *fosA3*, *bla*_CTX-M-65_, *tet(A)*, *dfrA12*, *aadA2*, *cmlA1*, *aadA1*, *qacL*, *sul3*	*bla*_CTX-M-65_

CABA, Ciudad Autónoma de Buenos Aires.

Hybrid assembly of short plus long reads was performed with Unicycler v0.4.8-beta. Open reading frames were annotated with PROKKA and manually curated. Sequence types using multilocus sequence typing (MLST) Achtman scheme was determined through MLST 2.0 (https://cge.food.dtu.dk/services/MLST/). PlasmidFinder, ResFinder (https://www.genomicepidemiology.org/services/), and ISFinder (https://isfinder.biotoul.fr/) were used to identify incompatibility groups, resistance genes and insertion sequences, respectively. Sequence comparisons were performed with Nucleotide BLAST, using the National Center for Biotechnology Information (NCBI) Nucleotide Collection Database, and Artemis Comparative Tool (ACT).

## Results

Eight *mcr-1*-harboring plasmids selected from a national collection of 192 *mcr-1*-positive *E*. *coli* clinical isolates from Argentina (Faccone, RPSP.2020), were subjected to whole genome sequencing. This selection included: four IncI2- and IncX4-negative isolates from Mendoza (1), La Pampa (1), and Buenos Aires City (2), and three IncX4-positive strains from Jujuy (1) and Buenos Aires City (2). In addition, even when some IncI2 plasmids from our country were previously sequenced, one IncI2-positive isolate from Cordoba (1) province, was also included because it was recovered more recently (July 2017). The genomic information of these eight *mcr-1*-positive *E*. *coli* isolates were summarized in Table 1.

MLST analysis revealed that three *E*. *coli* isolates belonged to ST131 high-risk clone, while the remaining five were singletons of ST38, ST46, ST226, ST224, and ST405 (Table 1). All *mcr-1* genes were found in closed plasmids sized between 33,304 bp and 274,233 bp and with one of the following replicase types: IncI2, IncX4, IncHI2/2A and FIA/HI1A/HI1B (Table 1). One isolate, namely M22546 (ST131), harbored *mcr-1*.*5* as a single acquired resistance gene, while the remaining seven isolates showed up to 14 acquired resistance genes (Table 1). Six isolates carried three allelic variants codifying for CTX-M-type extended-spectrum-β-lactamases (ESBLs): *bla*_CTX-M-2_ (3), *bla*_CTX-M-65_ (2), and *bla*_CTX-M-14_ (1) (Table 1).

### *mcr-1*-containing IncI2 plasmid

*E*. *coli* M22546 was one of the three isolates belonging to ST131 and harbored pEco_M22546_62, which was an IncI2 plasmid sizing 62,883bp (Table 1). Two copies of IS*Apl1* in the same orientation flanking *mcr-1*.*5* and *pap2* genes were found, as well as a GA target site duplication, strongly supporting the insertion of the composite transposon Tn*6330* [8]. Except for minor differences in the shufflon region, pEco_M22546_62 shared 100% identity with pMCR-M21015 and 99.97% with pMCR-M15049 plasmids, both recovered from multidrug resistant *Citrobacter amalonaticus* and *E*. *coli* clinical isolates, respectively, reported in Argentina [18, 20]. No additional resistance genes were observed in pEco_M22546_62, as was previously described for *mcr-1*-harboring IncI2-type plasmids [10, 18].

### *mcr-1*-containing IncX4 plasmids

*E*. *coli* isolates M23370 (ST38), M23059 (ST226) and M23917 (ST46) harbored *mcr-1*.*1* in the IncX4 plasmids pEco_M23370_33, pEco_M23059_33 and pEco_M23917_34, respectively, as a single antimicrobial resistance gene, as previously reported for other IncX4 *mcr-1*-harboring plasmids [13]. *E*. *coli* M23059 was recovered from blood sample in Jujuy province located 1,500 km apart from Buenos Aires City, where M23370 and M23917 were isolated from different hospitals (Table 1). These three isolates harbored two variants of CTX-M-type ESBL, and two of them showed resistance to other antimicrobial agents, besides colistin and β-lactams (Table 1). pEco_M23370_33, pEco_M23059_33 and pEco_M23917_34 carried *mcr-1*.*1* in a Tn*6330* remnant structure, lacking both IS*ApI1* copies. The three IncX4 plasmids showed 99.99% identity among them. pEco_M23917_34 also contained an IS*Kpn*26 inserted 61 bp upstream to *mcr-1*.*1*. The presence of IS*Kpn26* inside Tn*6330* was occasionally described but, to the best of our knowledge, this is the first report of this genetic platform in IncX4 plasmids [24]. The three IncX4 plasmids described here showed 99.96% identity and 100% of coverage with pCSZ4 (NCBI accession number KX711706) recovered from different bacterial species and sample origins worldwide [5, 13, 25].

### *mcr-1*-containing IncHI2/HI2A plasmids

Isolates M21816 and M23312, both ST131, were recovered from two hospitals located in Buenos Aires City, while M21170 (ST405) was isolated from Mendoza province (1,000 km apart). These three isolates harbored the alleles *mcr-1*.*1*, or *mcr-1*.*5*, in IncHI2/HI2A plasmids, named pEco_M21816_274 (274,233 bp), pEco_M23312_272 (272,608 bp) and pEco_M21170_247 (247,558 bp), respectively (Table 1).

**Fig 1:**
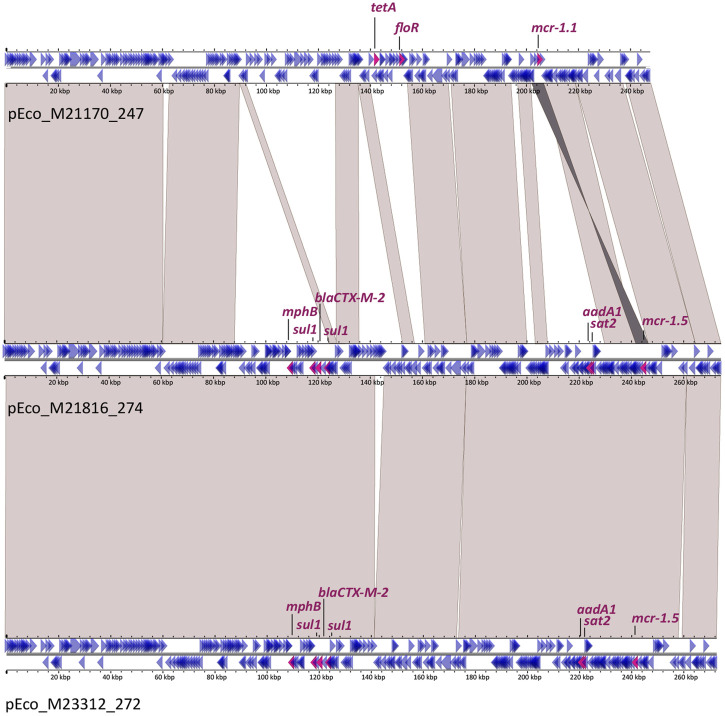
Sequence alignment of IncHI2/HI2A plasmids. pEco_M21170_247, pEco_M21816_274 and pEco_M23312_272. Genes are represented by arrows that indicate their transcriptional sense: magenta arrows for antimicrobial resistance genes (corresponding names are indicated) and blue arrows for other genes. Light grey shadows represent >99% identity between nucleotide sequences, and the dark grey shadow indicates inversion.

Both plasmids from *E*. *coli* ST131 isolates, pEco_M21816_274 and pEco_M23312_272, harbored *mcr-1*.*5* in Tn*6330* flanked by an AG target site duplication. Sequence comparison showed that these plasmids were essentially identical, differing only by the insertions of IS*1R* and IS*SBo1*, in pEco_M23312_272 and by an additional fragment of 4,089 bp in pEco_M21816_274, which included an IS*3* (Fig 1). Both pEco_M21816_274 and pEco_M23312_272 contained the following additional antimicrobial resistance genes: *aadA1*, *mph(B)*, *sat2* and *bla*_CTXM-2_. In both plasmids, *bla*_CTXM-2_ gene was located in the variable region 2 of complex class 1 integrons, as previously described [26]. Interestingly, in both complex class 1 integrons, the 5’-conserved segment (5-´CS) and the variable region 1 (*i*.*e*., the cassette region) were lost, and had an insertion of IS*1R* instead, which could probably be involved in such rearrangements (Fig 1).

The third IncHI2/HI2A plasmid, pEco_M21170_247, harbored the allele *mcr-1*.*1* also located in Tn*6330*, which was flanked by a GA target site duplication. This plasmid contained the additional antimicrobial resistance genes *tet(A)* and *floR*. The sequence comparison with the other two IncHI2/HI2A plasmids described above showed that pEco_M21170_247 only covered 72% of pEco_M21816_274 with 99.98% identity (Fig 1). Unlike the other two isolates harboring *bla*_CTX-2_, isolate M21170 had *bla*_CTXM-14_ encoding gene located in the chromosome, associated to IS*Ecp1*, as reported previously [27]. To the best of our knowledge, although only three *mcr-1*-containing IncHI2/HI2A plasmids were previously described in the Americas [5], our work constitutes its first description in Argentina.

### *mcr-1*-containing hybrid IncFIA/HI1A/HI1B plasmid

*E*.*coli* M23314 (ST224) was recovered from a blood sample of a patient in La Pampa province, 650 Km apart from Buenos Aires City (CABA). Plasmid pEco_M23314_185 (185,802 bp) harbored the *mcr-1*.*5* allele in the Tn*6330* context, showing GT as target site duplication. FIA, HI1A and HI1B replicon groups were detected in this plasmid. Besides *mcr-1*.*5*, the following antimicrobial resistance genes were identified in this plasmid: *aadA2*, *aadA2b*, *cmlA1* and *sul3*.

**Fig 2:**
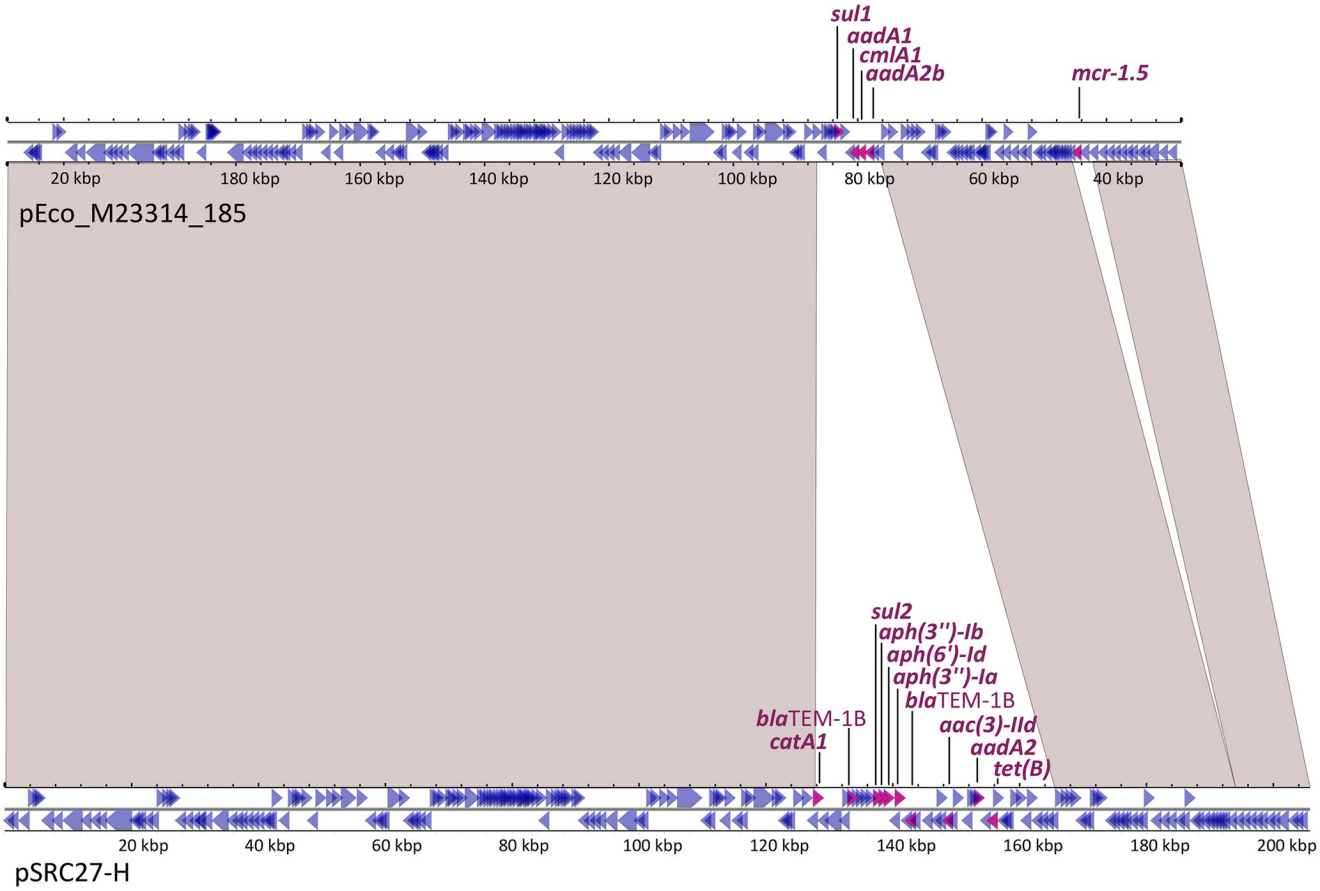
Sequence alignment between pEco_M23314_185 and pSRC27-H plasmids. Genes are represented by arrows that indicate their transcriptional sense: magenta arrows for antimicrobial resistance genes (corresponding names are indicated) and blue arrows for other genes. Light grey shadows represent >99% identity between nucleotide sequences.

The comparison of pEco_M23314_185 against the NCBI database (last accession April 1^st^ 2023) showed the highest score with pSRC27-H from *Salmonella enterica*, ser. Typhimurium recovered from an equine infection in Australia [28]. pEco_M23314_185 covered 92% of pSRC27-H (205,772bp) with 99.98% identity, but the latter lacked *mcr* genes or Tn*6330* remnant. pEco_M23314_185 showed the following differences with pSRC27-H: i) acquisition of Tn*6330*; ii) insertion of IS*Kpn74* downstream to *repB* of IncHI1B; and iii) a different gene rearrangement at the resistance region defined for pSRC27-H (Fig 2).

Therefore, we analyzed the first 5,000 hits with the highest scores and >25% coverage of pEco_M23314_185 that were obtained in the comparison of this plasmid against the NCBI database. Only 10 *mcr-1*-containing closed plasmids were found, but none of them harbored the *mcr-1*.*5* allele nor the antimicrobial resistance gene arrangement observed in pEco_M23314_185. The ten *mcr*-1-harboring plasmid sequences were from eight *E*. *coli*, one *S*. *enterica* and one *K*. *pneumoniae*. Considering pEco_M23314_185, the coverage of these 10 plasmids ranged between 80–96% and the sequence identity between 99.86–99.99% and showed the same FIA/HI1A/HI1B replicon combination. Of note, two plasmids, pSAL4596-1 and pCP53-mcr, co-harbored *mcr-1*.*1* with *mcr-5*.*1*, or *mcr-3*.*19*, respectively. Eight of these Enterobacterales were recovered between 2013 and 2016 from animals, animal foods or vegetable samples, in several countries of East and Southeast Asia (see S1 Table). Recently, two *mcr-1*-carrying hybrid plasmids were characterized in two *E*. *coli* isolates recovered from stool specimens of healthy residents from Ecuador [16]. These two hybrid plasmids, named pLR-06 and pLR-50 (260,770 bp and 197,729 bp, respectively), harbored the *mcr-1*.*1* allele and with another antimicrobial resistance gene combinations [16]. To the best of our knowledge, our work constitutes the first worldwide report of an *mcr-1*-containing hybrid FIA/HI1A/HI1B plasmid recovered from an isolate causing human infection.

## Discussion

It has been proposed that *E*. *coli* isolates containing IncI2 and IncX4 *mcr-1*-harboring plasmids are the main responsible of the worldwide dissemination of acquired colistin resistance [9]. However, the *mcr-1* alleles, as part of Tn*6330*, have demonstrated a high capacity to mobilize to different plasmid backbones [8, 9]. Complete sequence analysis of *mcr-1*-harboring plasmids belonging to incompatibility groups other than IncI2 and IncX4 are still scarce. The present study includes the analysis of IncI2 and IncX4 plasmids but also four unusual plasmids belonging to IncHI2/HI2A and a hybrid IncFIA/HI1A/HI1B. Both IncI2 and IncX4 plasmids described here showed high identity with other plasmids of these groups previously reported, however, our work constitutes the first characterization of IncX4 plasmids from Argentina. In contrast, IncHI2/IncHI2A plasmids are larger in size and has the capacity to harbor several acquired resistance genes, such as *bla*_CTX-M-2_ found in both plasmids pEco_M21816_274 and pEco_M23312_272 described here. The dissemination of IncHI2/IncHI2A plasmids, harboring multiple resistance genes, has relevant epidemiological and clinical impact because it limits therapeutic options.

IncFIA/HI1A/HI1B hybrid plasmid harboring *mcr-1* gene is uncommon and were described in a few samples from livestock, vegetables and healthy people [16]. Hybrid plasmids broad the host species increasing their dissemination capacity, and give the potential to survive under different environments [16]. The finding of IncFIA/HI1A/HI1B hybrid plasmids in different animal, food and human sources highlights the interconnection among these biological settings that enables the cross dissemination of diverse antimicrobial resistance genes.

In previous studies from our country, 14% of human *E*. *coli* isolates harbored the *mcr-1*.*5* allele [23], while a higher proportion of it was observed in plasmids recovered from chicken and pigs [29, unpublished data]. *mcr-1*.*5* seems to be a frequent allele in Argentina, while, at a worldwide level, it was only described in Bolivia and Japan sporadically [30, 31]. These findings suggest that *mcr-1*.*5* might play a signature role in tracking the dissemination of *mcr-1* alleles among different sources in our country. In addition, we found here *mcr-1*.*5* in three different plasmid backbones, representing the first description of this allele in IncHI2/HI2A and FIA/HI1A/HI1B groups.

In this work, all *mcr-1* alleles were located in Tn*6330*, or remnants of this transposon that lost both copies of IS*Apl1*, regardless of the incompatibility groups of the plasmids that harbored these genes. Therefore, the Tn*6330*-based dissemination of *mcr-1* to different plasmid backbones supports the notion that the horizontal mobilization of this gene type occurred not only by conjugation but also through transposition events. The horizontal dissemination of *mcr-1* through different plasmid backbones enables the possibility to be harbored by high-risk clones, like *E*. *coli* ST131, as we found here. Moreover, the detection of IncI2 or IncHI2/HI2A plasmids in *E*. *coli* ST131 isolates warns about the threat to human health given the broad spectrum of infections that this high-risk clone causes and the capacity to acquire antimicrobial resistance genes [32].

In conclusion, this study provides new insights of *mcr-1* dissemination among *E*.*coli* clinical isolates in Argentina, being the first characterization of IncX4 plasmids from our country. Additionally, we described *mcr-1*.*5* variant in three different plasmid backbones, representing the first description of this allele in IncHI2/HI2A and IncFIA/HI1A/HI1B plasmid groups. These conclusions underscore the aim of this study, which is to provide more light into the *mcr-1* landscape in Argentina.

One of the limitations of this study is that six out of ten IncI2- and IncX4-negative isolates found in our previous study were not included in the current study due to the limited economical resources available. An additional study including those six isolates is in progress. In addition, to obtain a more complete *mcr-1* landscape dissemination in our region, further analysis are necessary to address conjugation frequency, plasmid copy-number and stability of the already characterized *mcr-1*-harboring plasmids. Finally, further researches under the One Health perspective of *mcr-1*-containing genetic platforms is mandatory to integrate and understand the current antimicrobial resistance scenario.

## Supporting information

S1 Table Epidemiological information and comparison of pEco_M23314_185 against 10 *mcr-1*-containing plasmids from NCBI database. (XLSX)
